# Supplementary material for: Autochthonous Apple Cultivars from the Campania Region (Southern Italy): Bio-Agronomic and Qualitative Traits
Source: Plants (Basel). 2023 Mar 3;12(5):1160. doi: 10.3390/plants12051160 (PMC10007192; doi:10.3390/plants12051160)
Supplement: Supplementary file 1 [file plants-12-01160-s001.zip › Figure S1.pdf]

Figure S1. Photographic materials of the thirty-one autochthonous apple cultivars from Campania region and two commercial cultivars. (1) 'Acquata'; (2) 'Agostinella Rossa'; (3) 'Aitaniello'; (4) 'Ambrosio'; (5) 'Ananassa'; (6) 'Arancio'; (7) 'Arito'; (8) 'Austegna', (9) 'Austina'; (10) 'Cannamela'; (11) 'Carne'; (12) 'Chianella'; (13) 'Cusanara'; (14) 'Fragola'; (15) 'Latte'; (16) 'Lazzarola'; (17) 'Martina'; (18) 'Paradiso'; (19) 'Parrochiana'; (20) 'Prete'; (21) 'Re'; (22) 'San Francesco'; (23) 'San Giovanni'; (24) 'San Nicola'; (25) 'Suricillo'; (26) 'Tenerella'; (27) 'Trumuntana'; (28) 'Tubiona'; (29) 'Vivo'; (30) 'Zampa di Cavallo'; (31) 'Zitella'; (32) 'Annurca Rossa del Sud'; (33) Golden B.

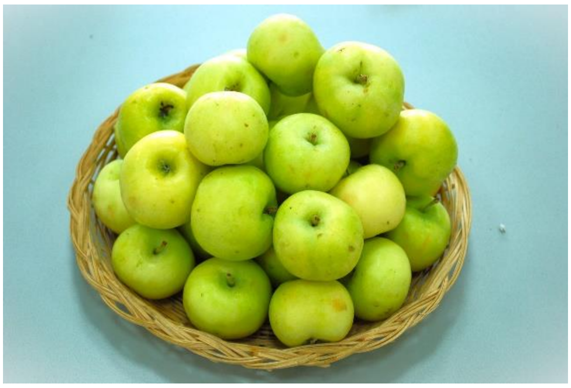

(1)

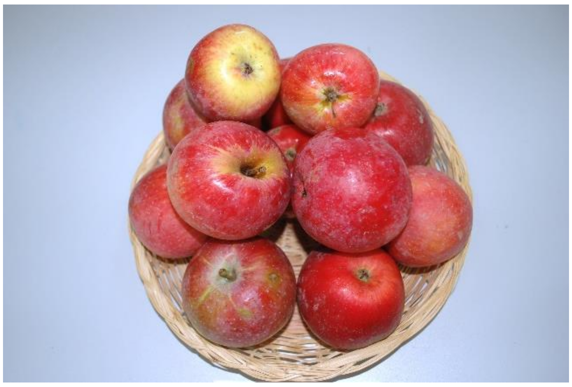

(2)

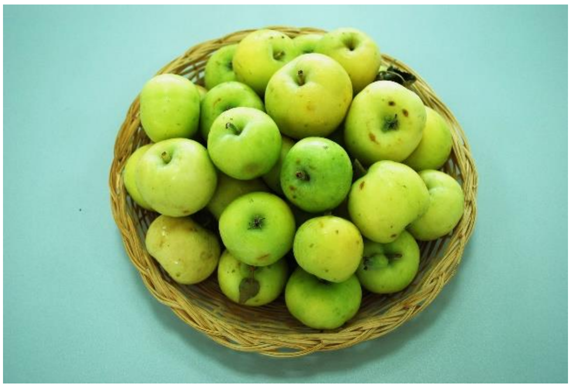

(3)

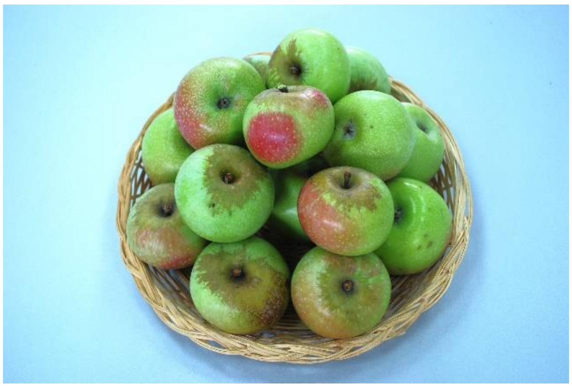

(4)

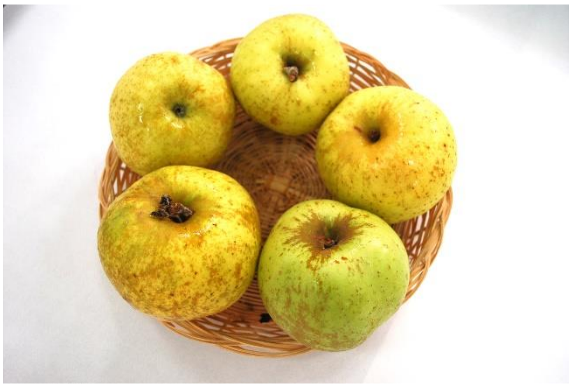

(5)

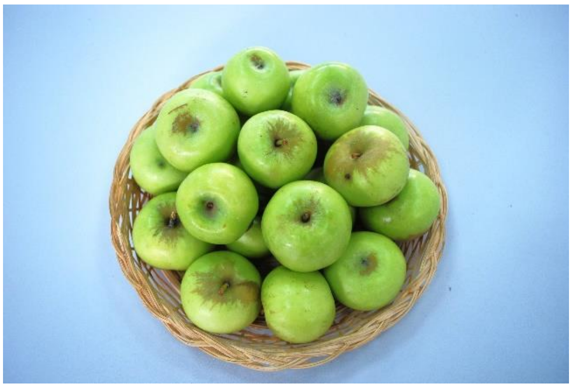

(6)

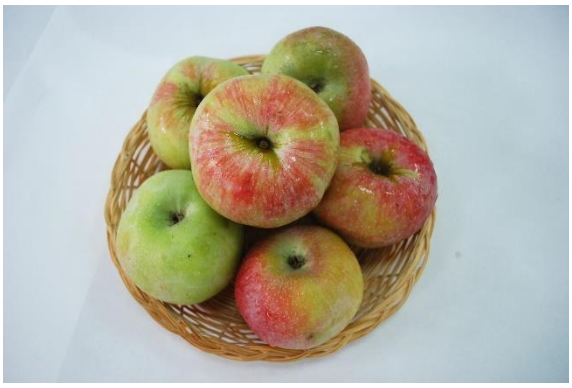

(7)

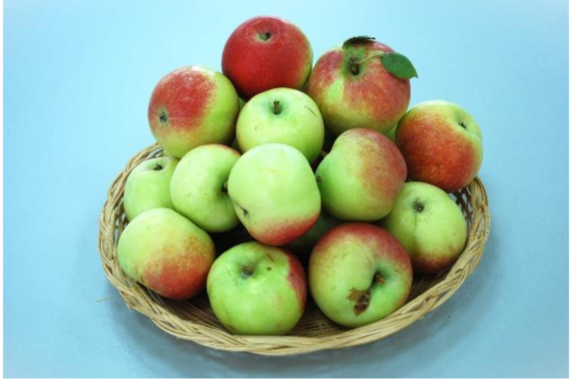

(8)

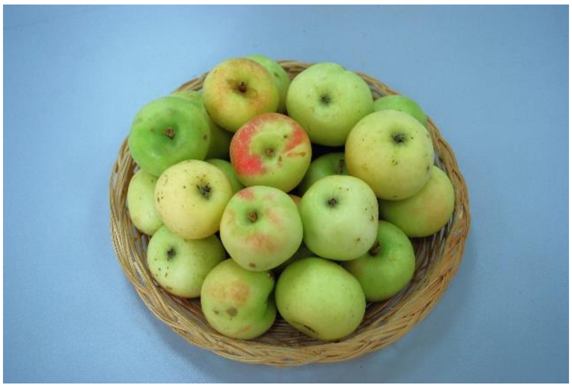

(9)

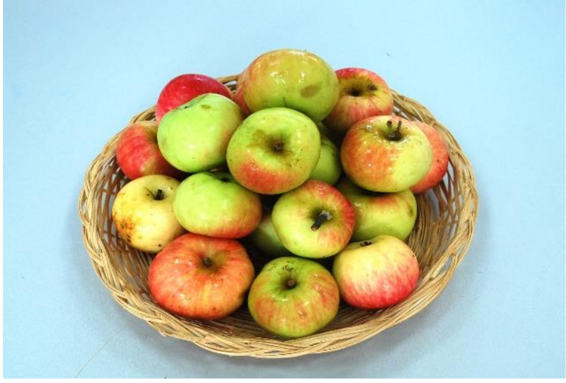

(10)

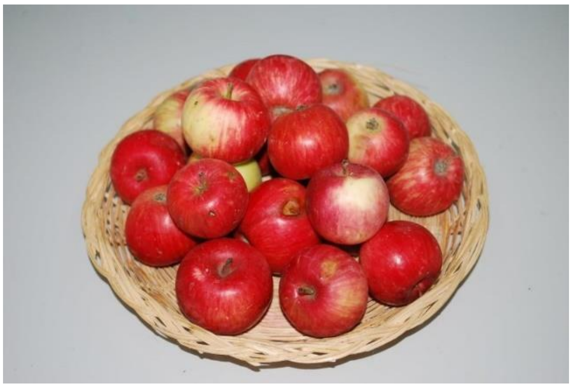

(11)

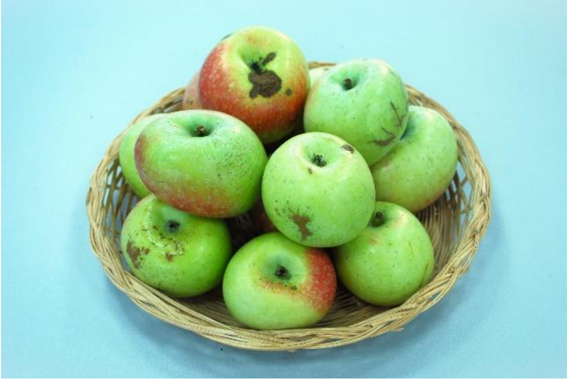

(12)

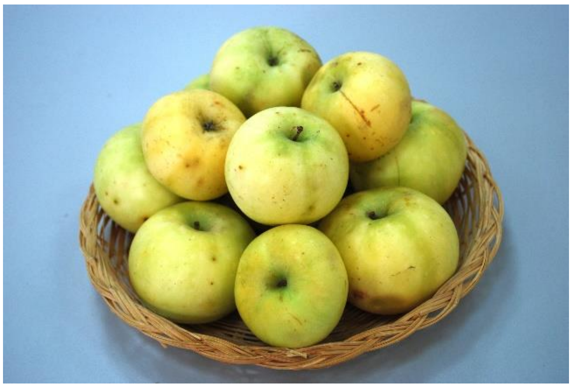

(13)

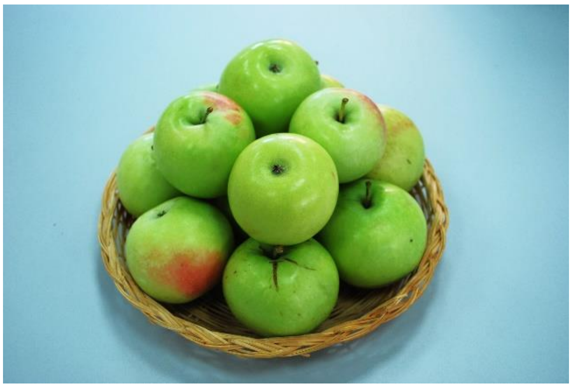

(14)

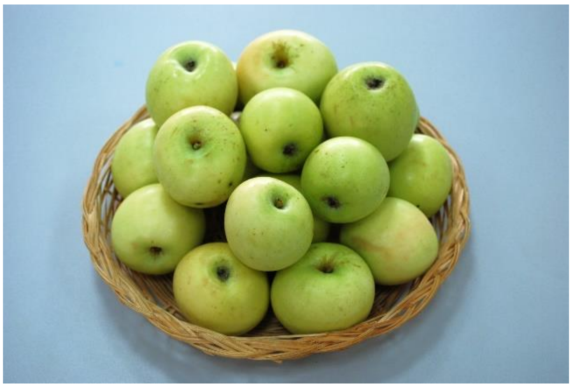
(15)

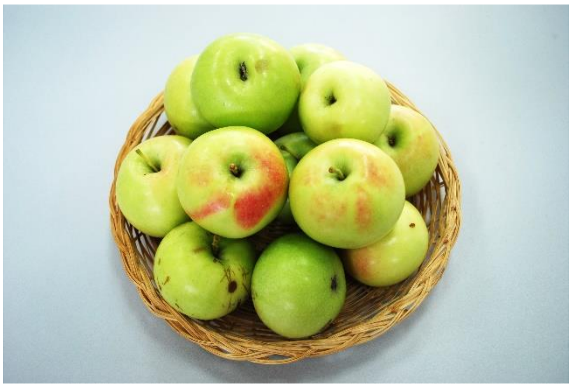
(16)

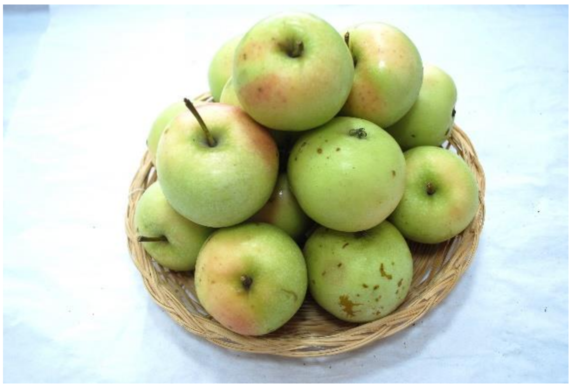
(17)

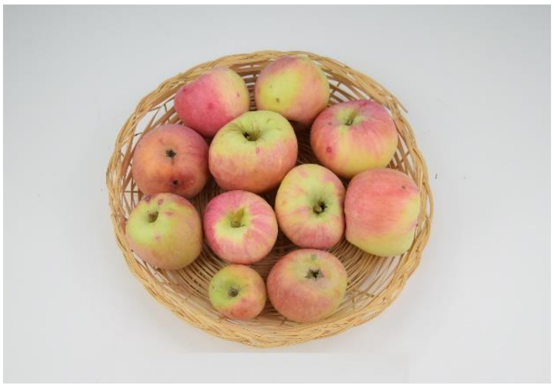
(18)

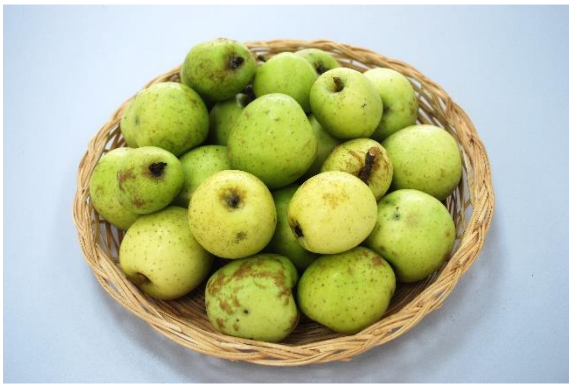
(19)

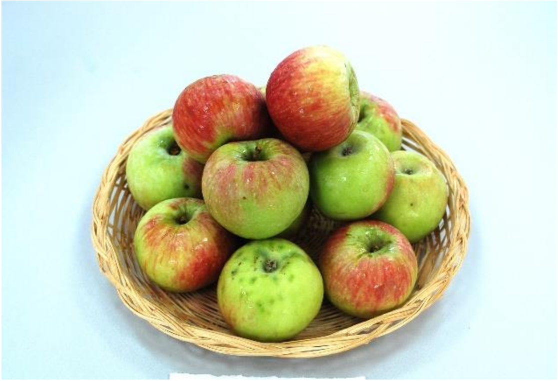
(20)

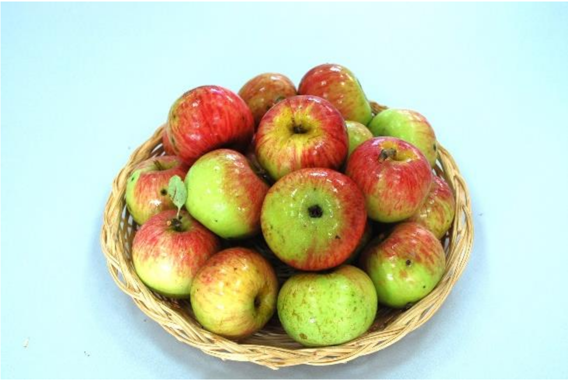
(21)

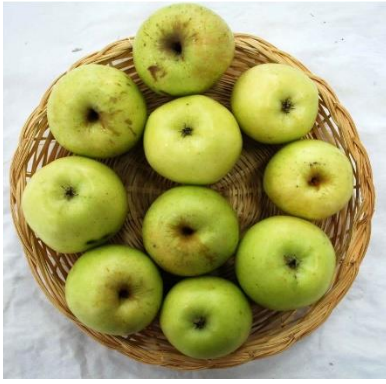
(22)

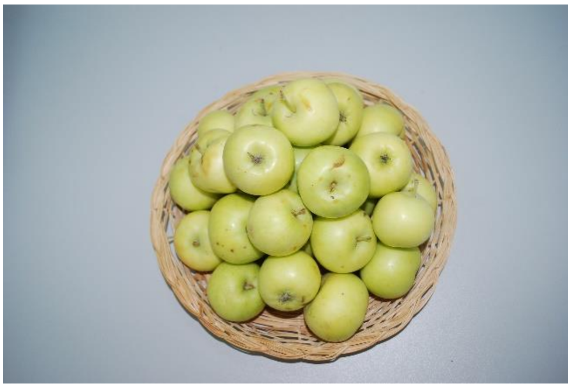

(23)

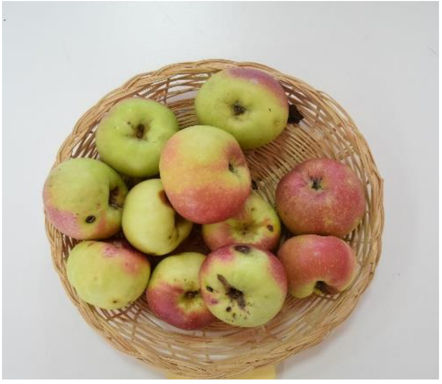

(24)

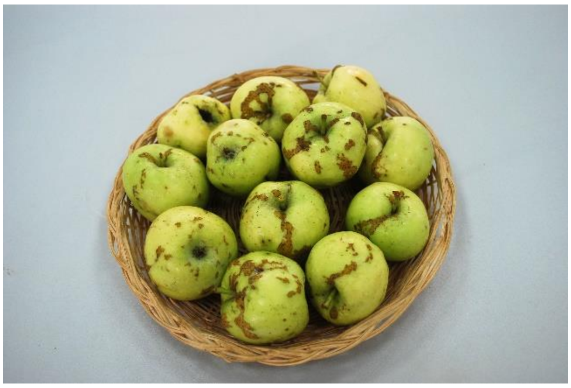

(25)

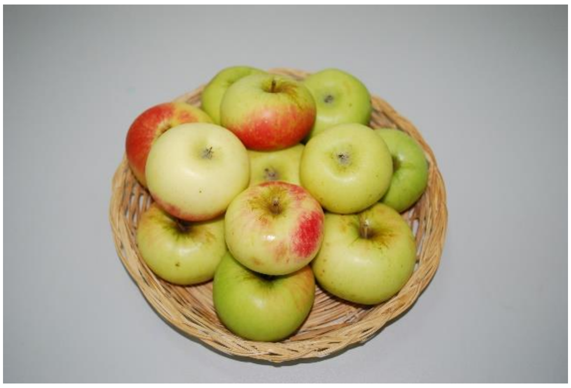

(26)

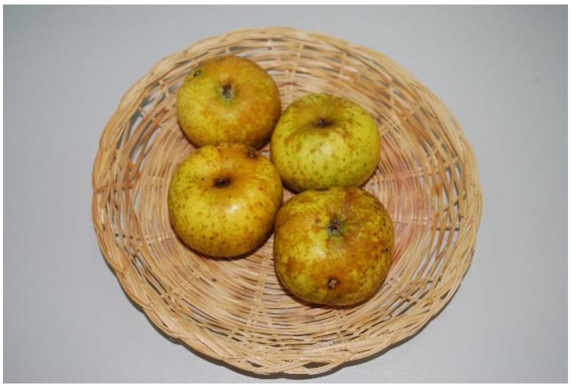

(27)

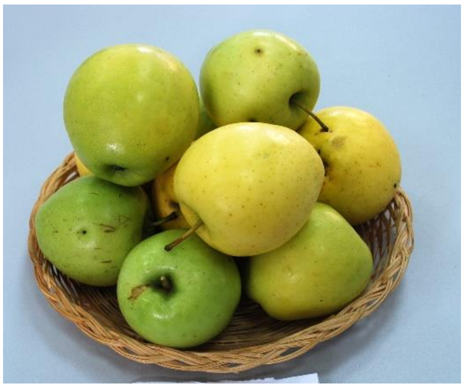

(28)

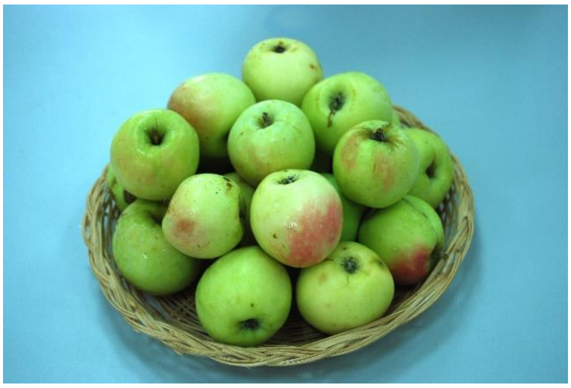

(29)

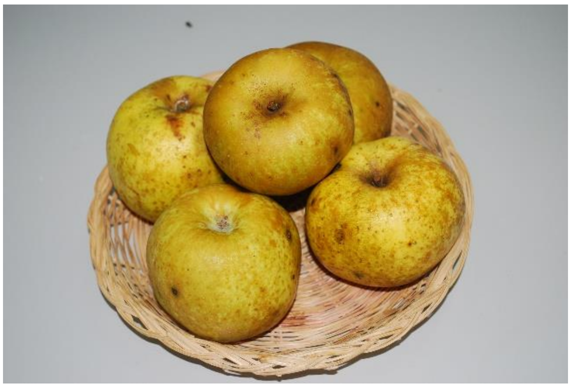

(30)

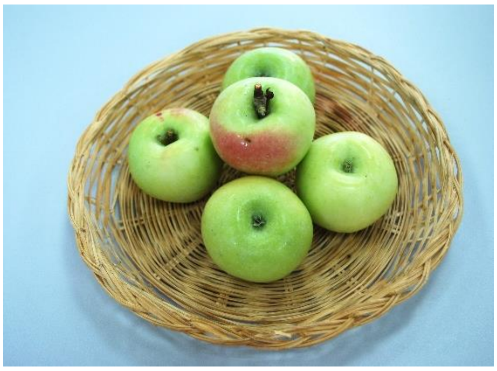

(31)

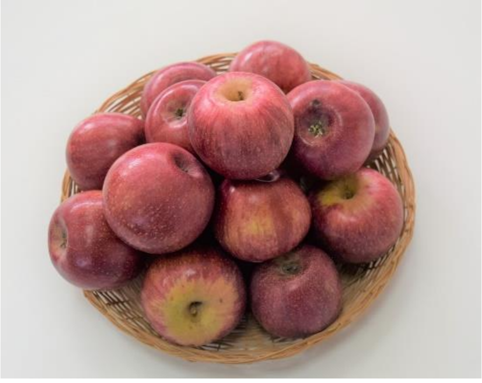

(32)

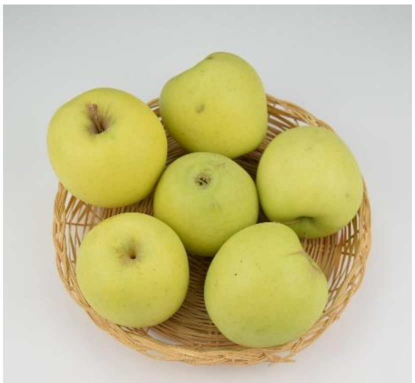

(33)
