# Supplementary material for: Autochthonous Apple Cultivars from the Campania Region (Southern Italy): Bio-Agronomic and Qualitative Traits
Source: Plants (Basel). 2023 Mar 3;12(5):1160. doi: 10.3390/plants12051160 (PMC10007192; doi:10.3390/plants12051160)
Supplement: Supplementary file 1 [file plants-12-01160-s001.zip › Table S1.pdf]

Table S1. UPOV descriptors and their abbreviations for apple.

	UPOV DESCRIPTORS	ABBREVIATION
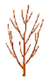	Tree: vigor	**V**
	Tree: type	**T**
	Only varieties with ramified tree type: Tree: habit	**TH**
	Tree: type of bearing	**TB**
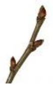	One year old shoot: thickness	**THI**
	One year old shoot: length of internodes	**L**
	One year old shoot: color on sunny side	**C**
	One year old shoot: pubescence	**P**
	One year old shoot: number of lenticels	**N**
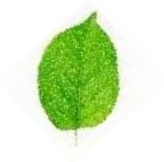	Leaf blade: attitude in relation to shoot	**A**
	Leaf blade: length	**LE**
	Leaf blade: width	**W**
	Leaf blade: ratio length/width	**R**
	Leaf blade: intensity of green color	**I**
	Leaf blade: incisions of margin	**IM**
	Leaf blade: pubescence on lower side	**PU**
	Petiole: length	**PL**
	Petiole: extent of anthocyanin coloration from base	**PA**
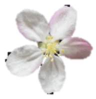	Flower: predominant color at balloon stage	**PC**
	Flower: diameter with petals pressed into horizontal position	**D**
	Flower: arrangement of petals	**AP**
	Flower: position of stigmas relative to anthers	**PS**
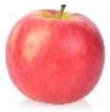	Young fruit: extent of anthocyanin overcolor	**Y**
	Fruit: size	**S**
	Fruit: height	**H**
	Fruit: diameter	**DI**
	Fruit: ratio height/diameter	**RH**
	Fruit: generalshape	**G**
	Fruit: ribbing	**RI**
	Fruit: crowning at calyx end	**CC**
	Fruit: size of eye	**SE**
	Fruit: length of sepal	**LS**
	Fruit: bloom of skin	**BS**
	Fruit: greasiness of skin	**GS**
	Fruit: ground color	**GC**
	Fruit: relative area of over color	**RA**
	Fruit: hue of over color with bloom removed	**HC**
	Fruit: intensity of over color	**IO**
	Fruit: pattern of over color	**PO**
	Fruit: width of stripes	**WS**
	Fruit: area of russet around stalk attachment	**AS**

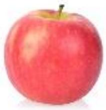	Fruit: area of russet on cheeks	**AC**
	Fruit: area of russet around eye basin	**AE**
	Fruit: number of lenticels	**NL**
	Fruit: size of lenticels	**SL**
	Fruit: length of stalk	**LES**
	Fruit: thickness of stalk	**TS**
	Fruit: depth of stalk cavity	**DS**
	Fruit: width of stalk cavity	**WC**
	Fruit: dept of eye basin	**DE**
	Fruit: width of eye basin	**WE**
	Fruit: firmness of flesh	**F**
	Fruit: color of flesh	**CF**
	Fruit: aperture of locules	**AL**
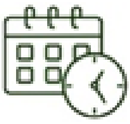	Time of beginning of flowering	**TF**
	Time for harvest	**THA**
	Time of eating maturity	**TE**
