# Supplementary material for: Autochthonous Apple Cultivars from the Campania Region (Southern Italy): Bio-Agronomic and Qualitative Traits
Source: Plants (Basel). 2023 Mar 3;12(5):1160. doi: 10.3390/plants12051160 (PMC10007192; doi:10.3390/plants12051160)
Supplement: Supplementary file 1 [file plants-12-01160-s001.zip › Table S2.pdf]

Table S2. Descriptors according to UPOV guidelines for apple cultivars

Cultivar	Tree				One-year-old shoot					Leaf blade									Flower			
CV	1	2	3	4	5	6	7	8	9	10	11	12	13	14	15	16	17	18	19	20	21	22
Acquata	5	2	1	3	3	5	3	3	3	1	5	5	7	5	2	2	3	5	5	5	2	3
Agostinella rossa	5	2	2	1	3	5	2	5	3	1	3	3	5	5	3	1	7	7	3	5	1	3
Aitaniello	5	2	1	2	3	5	3	3	3	1	5	5	7	5	4	2	5	5	5	5	2	3
Ambrosio	5	2	2	1	5	3	2	7	3	3	1	3	5	5	2	2	3	3	3	5	1	1
Ananassa	7	2	2	1	5	5	2	7	3	1	5	7	3	5	4	2	5	3	4	5	1	2
Arancio	5	2	1	2	5	5	2	7	5	1	7	5	7	5	2	2	7	3	4	5	2	2
Arito	5	2	1	2	3	5	2	3	5	1	7	3	7	5	2	1	7	3	3	5	1	3
Austegna	7	2	1	3	5	3	2	3	3	1	5	3	7	5	3	1	7	3	1	5	2	3
Austina	5	2	1	3	3	3	2	5	3	1	3	3	7	5	4	1	5	5	3	5	2	2
Cannamela	7	2	1	2	3	5	2	3	3	1	1	3	3	5	4	1	3	3	3	5	3	2
Carne	5	2	2	3	3	3	2	5	3	1	3	3	7	5	4	3	3	7	3	5	3	3
Chianella	7	2	1	3	5	5	4	5	3	1	3	3	7	5	4	2	5	5	4	5	1	3
Cusanara	5	2	2	3	3	5	4	5	3	1	1	3	7	5	4	1	5	3	4	5	2	2
Fragola	5	2	1	2	5	5	4	5	5	1	7	5	7	5	2	2	7	3	3	5	1	3
Latte	5	2	1	1	3	5	2	1	3	1	3	3	7	5	3	2	5	3	3	5	2	3
Lazzarola	5	2	1	1	3	5	2	7	3	1	5	5	5	5	3	1	7	3	3	5	1	1
Martina	5	2	2	3	5	5	2	5	5	1	3	3	7	5	2	1	3	3	1	5	2	3
Paradiso	5	2	2	3	3	5	2	5	3	1	1	3	5	5	2	1	3	3	3	5	2	2
Parrocchiana	5	2	1	3	9	3	3	3	3	1	5	5	5	5	4	1	7	3	3	5	2	1
Prete	5	2	1	1	3	5	2	7	5	1	3	3	7	5	4	2	5	5	3	5	1	3
Re	5	2	1	2	3	5	2	5	7	1	1	3	3	5	1	2	3	3	3	5	2	3
San Francesco	7	2	3	2	3	5	2	3	3	1	3	5	5	5	2	1	3	3	3	5	1	2
San Giovanni	5	2	1	2	3	1	3	3	3	1	5	5	7	5	4	2	5	5	5	5	2	3
San Nicola	5	2	2	2	5	5	2	5	5	1	3	5	5	3	2	1	3	3	3	5	1	2
Suricillo	5	2	1	2	5	7	2	5	5	1	7	7	7	5	3	2	7	3	1	7	1	3
Tenerella	5	2	2	3	5	3	2	3	5	1	7	7	5	5	3	1	7	7	3	5	3	3
Trumuntana	7	2	2	3	5	5	2	7	3	1	5	7	3	5	4	2	5	3	4	5	1	2
Tubiona	7	2	3	2	7	3	2	3	5	2	7	5	5	7	4	2	7	3	4	5	1	3
Vivo	5	2	1	3	5	5	3	3	3	1	3	5	5	5	2	1	5	3	3	5	2	2
Zampa di cavallo	7	2	2	3	5	5	2	7	3	1	5	7	3	5	4	2	5	3	4	5	1	2
Zitella	7	2	1	3	5	5	2	5	5	1	5	5	5	5	3	2	3	5	3	5	3	1
Annurca Rossa del Sud	7	2	2	2	7	5	2	3	5	1	5	5	5	5	5	2	7	3	4	5	2	2
Golden B	5	2	2	2	5	7	4	3	7	2	5	7	5	3	4	1	7	3	4	5	1	3

Cultivar	Fruit																															Time of beginning of flowering	Time for harvest	Time of eating maturity
CV	23	24	25	26	27	28	29	30	31	32	33	34	35	36	37	38	39	40	41	42	43	44	45	46	47	48	49	50	51	52	53	54	55	56	57
Acquata	1	1	3	3	5	7	1	1	5	3	2	2	5	1					1	1	1	3	3	3	5	3	5	3	5	5	1	3	3	1	1
Agostinella rossa	1	4	5	5	5	2	1	1	5	3	3	2	4	7	3	5	2	7	2	1	1	5	5	5	7	7	3	3	5	5	1	1	5	5	4
Aitaniello	1	1	3	3	5	2	1	1	5	3	2	2	5	1					1	1	1	3	3	3	5	5	5	5	5	7	1	3	3	1	1
Ambrosio	1	2	3	5	1	7	1	1	3	3	2	2	4	5	2	5	3	5	3	2	1	5	5	3	7	7	3	5	7	9	1	1	7	9	8
Ananassa	1	9	5	7	1	7	1	1	7	3	1	1	5	1		3	1		3	3	3	7	7	3	5	7	7	5	3	5	2	1	7	7	7
Arancio	1	3	3	3	7	6	1	1	7	3	2	2	5	1					1	1	1	3	3	3	5	5	7	5	5	7	1	2	9	9	9
Arito	1	9	5	5	5	2	1	1	7	3	2	1	4	5	3	5	2	5	2	1	1	7	3	3	7	3	7	3	7	9	1	2	5	7	7
Austegna	1	2	3	5	1	7	1	1	3	3	1	1	5	5	3	5	1		1	1	1	5	3	3	5	5	3	5	5	5	1	1	5	5	5
Austina	1	1	3	3	1	6	1	1	5	3	2	2	5	3	2	3	1		1	1	1	3	5	1	5	3	3	3	3	7	1	2	5	1	3
Cannamela	1	1	3	5	1	6	1	1	5	5	1	1	2	1	2	5	2	5	2	1	1	3	3	3	5	3	3	5	5	7	1	3	7	7	8
Carne	1	1	3	3	1	7	1	1	3	3	1	1	5	7	3	5	1	5	1	1	1	5	3	3	7	3	3	3	3	3	1	1	7	5	5
Chianella	3	2	3	5	1	7	1	1	5	3	2	1	5	1	3	5	2	3	1	1	1	3	7	3	5	5	3	5	7	7	1	1	7	7	9
Cusanara	1	2	3	3	1	7	1	2	3	3	2	1	4	1	2	3	2	3	1	1	1	3	3	3	5	3	3	3	3	5	1	2	7	3	3
Fragola	1	4	5	5	5	2	1	1	5	3	1	2	5	5	2	5	1		1	1	1	3	5	5	5	5	3	3	5	7	1	1	3	5	5
Latte	1	1	5	3	9	4	1	1	5	3	1	2	4	1					2	1	1	3	3	3	3	5	3	5	3	5	1	2	7	7	7
Lazzarola	1	4	5	7	7	6	1	2	5	5	1	2	6	3	2	3	6		1	1	1	5	3	5	3	5	3	7	3	5	2	1	7	5	5
Martina	1	5	5	5	7	6	1	1	3	5	1	1	5	1	2	3	1		1	1	1	7	3	7	5	3	3	3	3	5	1	1	7	5	5
Paradiso	1	3	5	5	5	2	1	2	5	3	1	1	5	7	3	5	4	7	2	1	1	5	5	5	5	5	5	5	5	7	1	1	5	7	7
Parrocchiana	1	4	5	3	9	5	1	1	3	3	1	1	5	1					1	1	1	5	3	3	7	5	3	5	5	5	1	1	7	7	9
Prete	3	2	3	5	1	7	1	1	3	3	2	1	5	7	3	5	3	5	1	1	1	3	5	3	5	3	3	5	3	5	1	1	5	9	5
Re	1	1	3	5	1	7	1	1	5	3	1	1	4	5	3	5	7	5	2	1	1	3	3	3	5	3	3	3	5	9	1	1	5	5	7
San Francesco	1	5	7	7	7	2	1	2	7	3	1	1	5	1					1	1	1	5	7	7	5	5	5	5	5	7	1	1	5	7	7
San Giovanni	1	1	3	3	5	2	1	1	5	3	2	2	5	1					1	1	1	3	3	3	5	3	5	3	5	7	1	3	3	1	1
San Nicola	3	1	5	5	3	7	1	1	5	3	1	1	5	7	3	5	6		1	1	1	5	7	1	5	7	3	7	3	9	1	1	5	7	8
Suricillo	1	1	3	3	9	4	2	2	3	3	1	1	5	1					2	2	2	3	3	3	3	3	3	3	3	7	1	1	5	7	8
Tenerella	1	5	3	5	1	7	1	1	3	3	1	1	5	5	3	5	1		1	1	1	5	7	5	5	5	5	5	3	3	1	1	5	7	9
Trumuntana	1	9	5	5	1	7	1	1	7	3	1	1	5	1		3	1		3	3	3	7	7	3	5	7	7	5	3	5	2	2	7	7	7
Tubiona	1	9	7	7	7	2	1	2	7	3	1	1	5	1					1	1	1	5	7	7	5	5	5	5	5	3	1	2	5	5	6
Vivo	1	1	3	3	7	6	1	1	3	3	1	1	5	1	2				1	1	2	7	3	3	3	7	5	5	3	7	1	1	5	7	7
Zampa di cavallo	1	9	5	7	1	7	1	1	7	3	1	1	5	1		3	1		3	3	3	7	7	3	5	7	7	5	3	5	2	2	7	7	7
Zitella	1	1	5	5	5	2	1	2	3	3	1	1	5	3	3	3	1		1	1	1	3	3	5	5	5	5	5	3	5	1	1	7	7	7
Annurca Rossa del Sud	9	5	5	7	5	7	1	1	7	5	1	1	5	9	3	5	3	3	1	1	3	7	7	1	5	7	3	3	7	7	1	2	5	7	8
Golden B	1	9	7	5	7	2	2	2	7	7	1	1	5	1					1	1	1	5	3	7	3	5	5	5	5	7	2	2	5	7	7
