# Supplementary material for: Autochthonous Apple Cultivars from the Campania Region (Southern Italy): Bio-Agronomic and Qualitative Traits
Source: Plants (Basel). 2023 Mar 3;12(5):1160. doi: 10.3390/plants12051160 (PMC10007192; doi:10.3390/plants12051160)
Supplement: Supplementary file 1 [file plants-12-01160-s001.zip › Table S3.pdf]

Table S3. Frequency distribution of numerical note for each UPOV descriptors in autochthonous apple cultivars compared to standard cultivar ('Annurca Rossa del Sud' and 'Golden B').

	Numerical notes								
**UPOV descriptors**	1	2	3	4	5	6	7	8	9
**1**	0.0	0.0	0.0	0.0	69.7	0.0	30.3	0.0	0.0
**2**	0.0	100.0	0.0	0.0	0.0	0.0	0.0	0.0	0.0
**3**	54.5	39.4	6.1	0.0	0.0	0.0	0.0	0.0	0.0
**4**	18.2	39.4	42.4	0.0	0.0	0.0	0.0	0.0	0.0
**5**	0.0	0.0	45.5	0.0	45.5	0.0	6.1	0.0	3.0
**6**	3.0	0.0	21.2	0.0	69.7	0.0	6.1	0.0	0.0
**7**	0.0	72.7	15.2	12.1	0.0	0.0	0.0	0.0	0.0
**8**	3.0	0.0	39.4	0.0	36.4	0.0	21.2	0.0	0.0
**9**	0.0	0.0	60.6	0.0	33.3	0.0	6.1	0.0	0.0
**10**	90.9	6.1	3.0	0.0	0.0	0.0	0.0	0.0	0.0
**11**	15.2	0.0	30.3	0.0	36.4	0.0	18.2	0.0	0.0
**12**	0.0	0.0	42.4	0.0	39.4	0.0	18.2	0.0	0.0
**13**	0.0	0.0	15.2	0.0	39.4	0.0	45.5	0.0	0.0
**14**	0.0	0.0	6.1	0.0	90.9	0.0	3.0	0.0	0.0
**15**	3.0	30.3	21.2	42.4	3.0	0.0	0.0	0.0	0.0
**16**	45.5	51.5	3.0	0.0	0.0	0.0	0.0	0.0	0.0
**17**	0.0	0.0	30.3	0.0	33.3	0.0	36.4	0.0	0.0
**18**	0.0	0.0	69.7	0.0	21.2	0.0	9.1	0.0	0.0
**19**	9.1	0.0	54.5	27.3	9.1	0.0	0.0	0.0	0.0
**20**	0.0	0.0	0.0	0.0	97.0	0.0	3.0	0.0	0.0
**21**	45.5	42.4	12.1	0.0	0.0	0.0	0.0	0.0	0.0
**22**	12.1	36.4	51.5	0.0	0.0	0.0	0.0	0.0	0.0
**23**	87.9	0.0	9.1	0.0	0.0	0.0	0.0	0.0	3.0
**24**	36.4	15.2	6.1	12.1	12.1	0.0	0.0	0.0	18.2
**25**	0.0	0.0	48.5	0.0	42.4	0.0	9.1	0.0	0.0
**26**	0.0	0.0	33.3	0.0	48.5	0.0	18.2	0.0	0.0
**27**	39.4	0.0	3.0	0.0	27.3	0.0	21.2	0.0	9.1
**28**	0.0	30.3	0.0	6.1	3.0	18.2	42.4	0.0	0.0
**29**	93.9	6.1	0.0	0.0	0.0	0.0	0.0	0.0	0.0
**30**	75.8	24.2	0.0	0.0	0.0	0.0	0.0	0.0	0.0
**31**	0.0	0.0	33.3	0.0	39.4	0.0	27.3	0.0	0.0
**32**	0.0	0.0	84.8	0.0	12.1	0.0	3.0	0.0	0.0
**33**	66.7	30.3	3.0	0.0	0.0	0.0	0.0	0.0	0.0
**34**	69.7	30.3	0.0	0.0	0.0	0.0	0.0	0.0	0.0
**35**	0.0	3.0	0.0	18.2	75.8	3.0	0.0	0.0	0.0
**36**	54.5	0.0	9.1	0.0	18.2	0.0	15.2	0.0	3.0
**37**	0.0	24.2	36.4	0.0	0.0	0.0	0.0	0.0	0.0
**38**	0.0	0.0	24.2	0.0	42.4	0.0	0.0	0.0	0.0
**39**	30.3	15.2	9.1	3.0	0.0	6.1	3.0	0.0	0.0
**40**	0.0	0.0	9.1	0.0	18.2	0.0	6.1	0.0	0.0
**41**	66.7	21.2	12.1	0.0	0.0	0.0	0.0	0.0	0.0

**42**	84.8	6.1	9.1	0.0	0.0	0.0	0.0	0.0	0.0
**43**	81.8	6.1	12.1	0.0	0.0	0.0	0.0	0.0	0.0
**44**	0.0	0.0	42.4	0.0	36.4	0.0	21.2	0.0	0.0
**45**	0.0	0.0	54.5	0.0	18.2	0.0	27.3	0.0	0.0
**46**	9.1	0.0	60.6	0.0	18.2	0.0	12.1	0.0	0.0
**47**	0.0	0.0	15.2	0.0	69.7	0.0	15.2	0.0	0.0
**48**	0.0	0.0	33.3	0.0	42.4	0.0	24.2	0.0	0.0
**49**	0.0	0.0	54.5	0.0	30.3	0.0	15.2	0.0	0.0
**50**	0.0	0.0	36.4	0.0	57.6	0.0	6.1	0.0	0.0
**51**	0.0	0.0	45.5	0.0	42.4	0.0	12.1	0.0	0.0
**52**	0.0	0.0	9.1	0.0	39.4	0.0	39.4	0.0	12.1
**53**	84.8	15.2	0.0	0.0	0.0	0.0	0.0	0.0	0.0
**54**	57.6	30.3	12.1	0.0	0.0	0.0	0.0	0.0	0.0
**55**	0.0	0.0	12.1	0.0	45.5	0.0	39.4	0.0	3.0
**56**	12.1	0.0	3.0	0.0	24.2	0.0	51.5	0.0	9.1
**57**	9.1	0.0	6.1	3.0	18.2	3.0	33.3	15.2	12.1
