# Supplementary material for: Autochthonous Apple Cultivars from the Campania Region (Southern Italy): Bio-Agronomic and Qualitative Traits
Source: Plants (Basel). 2023 Mar 3;12(5):1160. doi: 10.3390/plants12051160 (PMC10007192; doi:10.3390/plants12051160)
Supplement: Supplementary file 1 [file plants-12-01160-s001.zip › Table S4.pdf]

Table S4. Analysis of Variance (ANOVA) for fruit weight (FW; g), firmness (F), solid soluble content (SSC), titratable acidity (TA), juice pH, Young's modulus (YM) and browning index (BI).

	FW	F	SSC	TA	pH	YM	BI
F	192.36	38.76	47.63	129.83	42.61	218.72	77.86
P	< 0.001	< 0.001	< 0.001	< 0.001	< 0.001	< 0.001	< 0.001
ω^2	0.903	0.537	0.938	0.977	0.931	0.986	0.961
